# Natal Teeth in an Infant With Down Syndrome: A Rare Presentation With a Genetic Evaluation and Review of the Literature

**DOI:** 10.7759/cureus.30101

**Published:** 2022-10-09

**Authors:** Abdullah Alassaf

**Affiliations:** 1 Preventive Dental Science, College of Dentistry, Majmaah University, Almajmaah, SAU

**Keywords:** trisomy of 21, vitamin k, infant, down's syndrome, natal teeth

## Abstract

Teeth at the time of birth are termed "natal teeth." The trisomy of the 21^st ^chromosome causes Down syndrome. Natal teeth in Down syndrome patients have not been reported frequently. The purpose of the present report was to describe a case of an infant with natal teeth and multiple cardiac problems, and karyotyping of his peripheral blood smear showed trisomy of the 21^st^ chromosome. Management of natal teeth and the rationale for natal teeth have been demonstrated. The novelty of the case report is that it reports the first Indian infant to report on natal teeth in Down syndrome patients.

## Introduction

Teeth present at birth are termed natal teeth, whereas teeth that erupt prior to 30 days after delivery have been considered neonatal teeth [1]. The natal or neonatal teeth are commonly observed in the anterior region of the mandibular arch [2,3]. They have a female predilection with a 1:3 ratio compared with males. The incidence of natal and neonatal teeth ranges from 1 in 2000 to 1 in 6000 live births [2]. Various etiological factors have been attributed to natal and neonatal teeth, and a clear explanation has not been reported [2]. Natal teeth are associated with genetic conditions such as Down's syndrome, Ellis Van Creveld syndrome, Hallermann-Strieff syndrome, pachyonychia congenital, cleft lip and palate, and cyclopia [3]. Health issues, including learning and memory, genetic heart diseases, Alzheimer's diseases, Hirschsprung's disease, and atypical facial features, are common in individuals with Down syndrome [4,5]. Down syndrome is a complex phenotype resulting from the dosage imbalance of genes located on human chromosome 21 [3,4]. The associations of natal teeth in Down syndrome infants have not been frequently reported in the literature. Therefore, the present report describes a case of a Down syndrome infant with natal teeth and gene karyotyping.

## Case presentation

**Figure 1:**
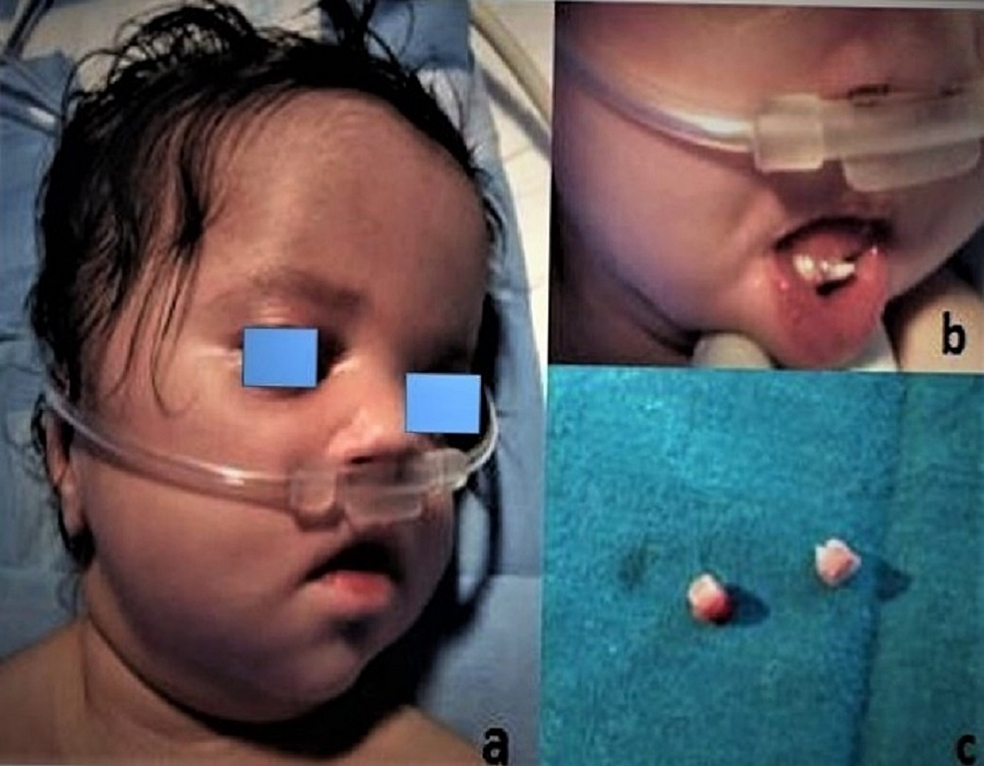
(a) Frontal view of an infant, (b) presence of mandibular natal teeth in a pre-term infant, and (c) showing extracted natal teeth without roots.

A five-day-old female neonate was referred to a pediatric dentist to manage erupted teeth at birth. She was the fourth child of a healthy non-consanguineous Islamic family. Her medical history showed that she was diagnosed with multiple congenital heart abnormalities. The baby girl was born full-term by vaginal delivery to a 40-year-old mother. Her birth weight was 1.97 kg (below the third percentile), and her height was about 35 cm (below the third percentile) (Figure 1a).

**Figure 2:**
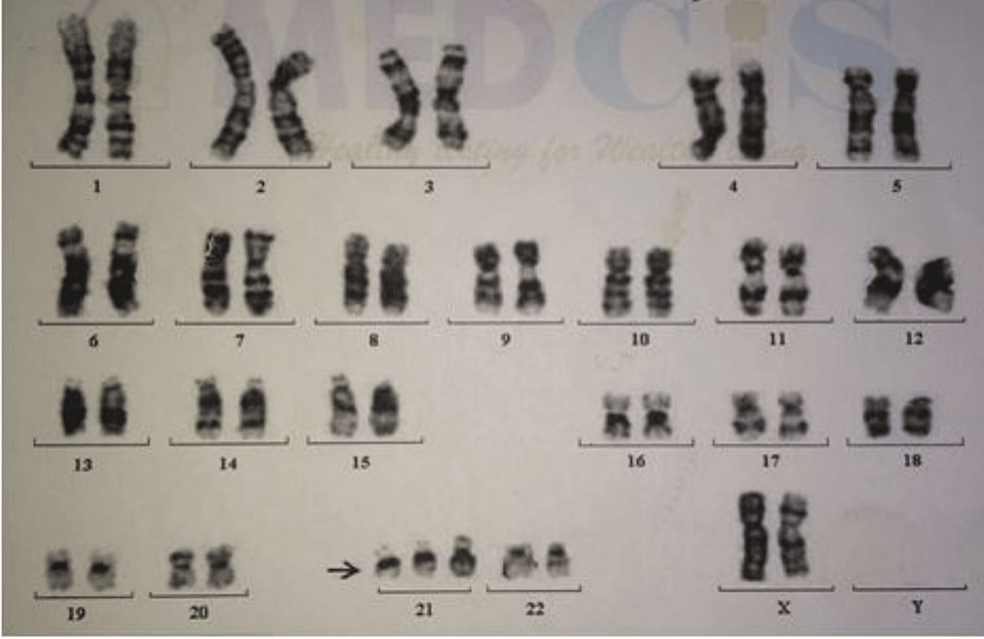
Karyogram of the male chromosomal complement with no numerical or structural chromosomal anomalies detected on 21st chromosome.

She was diagnosed with Down syndrome at the time of birth. Examination of her parents and siblings revealed that they were unaffected by the syndrome. Vitamin K (1.0 mg) was administered through the intramuscular route at the time of birth. Cytogenetic evaluation of phytohemagglutinin-stimulated peripheral blood lymphocytes revealed a trisomy of the 21st chromosome in standard condition (Figure 2).

Intra-orally, two natal teeth were observed in the mandibular arch (Figure 1b) at birth, and the teeth were mobile. None of the family members had a known history of teeth at birth. Consultation and approval from a pediatrician and pediatric cardiologist were obtained before extracting natal teeth. After discussing it with the parents, the natal teeth were removed under local anesthesia eight days after birth. Roots were absent in extracted natal teeth (Figure 1c).

## Discussion

In the mandibular arch, natal teeth are frequently observed and are more common in the anterior region [1,2]. The occurrence of natal and neonatal teeth is very often in the mandibular anterior region (85%), followed by the maxillary anterior region (11%), mandibular canine region (3%), and maxillary canine and molar regions (1%) [3]. The majority (90%) of these teeth are considered early erupted primary teeth, while the rest, 10% contemplated, are redundant [6]. The treatment options included extraction, composite splint, and grinding of sharp cusps, and if teeth, a mobile extraction of the natal teeth is recommended [1-6]. Trauma to newborns might cause bleeding, and to avoid bleeding in newborns, an intramuscular vitamin-K injection has been suggested [7]. Newborns tend to have vitamin-K deficiency due to inadequate stores at birth and deficient intake [7]. Classical vitamin-K deficiency bleeding arises between 24 hours and seven days after delivery and is also associated with delayed feeding [7]. It is recommended that newborns receive an intramuscular dose of 0.5 mg to 1.0 mg of vitamin K within the first six hours after birth [6,7]. However, 1 mg of vitamin K was administered at birth. Vitamin K is essential in synthesizing prothrombin in the liver, which is a critical component in blood clotting. The extraction of natal teeth was performed on the eighth day after birth. In the majority of the reported cases, natal or neonatal teeth cause lacerations on the ventral surface of the tongue (Rega feda disease) [2,6]. However, this pathology was not associated with natal teeth. The early extraction of mobile natal or neonatal teeth has been recommended [8]. In the present case, karyotyping of the peripheral blood smear showed trisomy of the 21st chromosome [9], diagnosing Down syndrome. The incidence of Down syndrome increases with maternal age from 1 in 319 to 1 in 1000 [10]. Similarly, in the present case, the child was the fourth baby, and the mother's age was 40 years old. This report serves as one of the examples explaining that late pregnancy will increase the chances of Down syndrome probability [11]. Furthermore, the patient exhibited multiple cardiac anomalies. Before this report, only three cases were reported of natal or neonatal teeth associated with Down syndrome. Among these three reported cases (Table 1), one each is from Nigeria [12], Sri Lanka [13], and the United States of America [2]. Natal teeth have not been reported frequently in association with Down syndrome. To the best of our knowledge, this is the only first case of an Indian subject reporting having natal teeth in Down syndrome patients.

**Table 1:** Published reports on Down syndrome with natal teeth.

Author	Year	Country	Gender	Age	Teeth	Remarks
Senanayake and Karunaratne [12]	2014	Sri Lanka	Male	18 months	71,81	Riga-Fede disease
Ndiokwelu et al. [13]	2004	Nigeria	Female	4 days	-	-
Mhaske et al. [2]	2000	United States of America	Male	10 months	71,81	Smoothen the teeth

## Conclusions

This case report serves as the first Indian infant to report natal teeth associated with Down syndrome. Early management of natal or neonatal teeth is essential to avoid potential complications. The role of vitamin K administration should be taken into consideration upon consultation with the pediatrician. A multispecialty management approach plays a key role in the management of natal and neonatal teeth.
